# Personal Exposure to Sulfuric Acid in the Electroplating Industry: Development and Validation of a Predictive Model

**DOI:** 10.3390/toxics12070489

**Published:** 2024-07-03

**Authors:** Austin B. Wang, Kai-Jen Chuang, Ven-Shing Wang, Ta-Yuan Chang

**Affiliations:** 1 Department of Occupational Safety and Health, College of Public Health, China Medical University, No. 100, Sec. 1, Jingmao Rd. Beitun Dist., Taichung City 406040, Taiwan; a93jb15@gmail.com (A.B.W.); vswang@mail.cmu.edu.tw (V.-S.W.); 2 Department of Public Health, College of Public Health, China Medical University, Taichung City 406040, Taiwan; 3 School of Public Health, College of Public Health, Taipei Medical University, No. 250, Wuxing St., Xinyi Dist., Taipei City 110, Taiwan; kjc@tmu.edu.tw; 4 Department of Public Health, School of Medicine, College of Medicine, Taipei Medical University, Taipei City 110, Taiwan

**Keywords:** electroplating, exposure assessment, personal exposure, predictive model, sulfuric acid

## Abstract

This study aimed to measure personal exposure to sulfuric acid in the electroplating industry to establish a predictive model and test its validation. We collected indoor air parameters and related information from four electroplating plants. Silica gel sorbents were used to collect air samples using high-performance ion chromatography. We collected air samples from three plants (i.e., Plant B, Plant C, and Plant D) and applied multiple linear regressions to build a predictive model. Eight samples collected from the fourth plant (i.e., Plant A) were used to validate the model. A total of 41 samples were collected with a mean of 25.0 ± 9.8 μg/m^3^ (range 12.1–51.7 μg/m^3^) in this study, including Plant A (8 samples, 17.5 ± 2.8 μg/m^3^, 13.0–22.0 μg/m^3^), Plant B (11 samples, 36.5 ± 9.7 μg/m^3^, 23.1–51.7 μg/m^3^), Plant C (11 samples, 16.4 ± 1.7 μg/m^3^, 12.1–17.8 μg/m^3^), and Plant D (11 samples, 27.4 ± 1.7 μg/m^3^, 24.1–29.9 μg/m^3^). Plant B was significantly higher in sulfuric acid than the other plants. Workers from the electroplating process plants were exposed to sulfuric acid at 29.0 ± 11.5 μg/m^3^. The predictive model for personal exposure to sulfuric acid fit the data well (r^2^ = 0.853; adjusted r^2^ = 0.837) and had an accuracy of 5.52 μg/m^3^ (bias ± precision; 4.98 ± 2.38 μg/m^3^), validated by the personal sampling of the fourth plant. This study observed that sulfuric acid exposure was lower than the permissible exposure level of 1000 μg/m^3^ in Taiwan and the United States, and only two samples were lower than the European Union standard of 50 μg/m^3^. The developed model can be applied in epidemiological studies to predict personal exposure to sulfuric acid in plants using electroplating.

## 1. Introduction

The International Agency for Research on Cancer (IARC) [1] has classified strong-inorganic-acid mists containing sulfuric acid as a Group 1 human carcinogen [1], specifically increasing the risk of laryngeal [2,3] and lung cancers [4]. In addition to its carcinogenic risk, strong-inorganic-acid mist also poses acute health risks, such as clinical and histopathological changes of the nasal mucosa [5], tooth erosion [6], and eye and skin chemical burns [7,8].

Many organizations have established standards to limit sulfuric acid exposure to improve workers’ health. However, these standards differ from various organizations. A permissible exposure limit (PEL) of 1 mg/m^3^ [9] proposed by the Occupational Safety and Health Administration (OSHA) in the United States is the same as the recommended exposure limit proposed by the National Institute for Occupational Safety and Health [10], as well as the PEL in Taiwan [11]. The European Chemicals Agency (ECHA) established an occupational exposure limit (OEL) of 0.05 mg/m^3^ for inhalable dust fractions due to the reason that it is a carcinogen [12]. The difference between the standards presents dissimilar attention to the issue, which confirms the importance of the related personal monitoring.

Many epidemiological studies have shown that sulfuric acid is widely used in diverse industries, including isopropanol, lead battery, and electroplating manufacturing [1]. However, studies on personal exposure to sulfuric acid in the electroplating process are relatively limited. The IARC report showed that the ratio of personal (n = 35) to area sampling (n = 154) was 0.25 [1], which shows that the number of personal-exposure-related studies has a relatively lower ratio. In an exposure study, the environmental monitoring levels in the electrolytic refining process were 0.01–5.6 mg/m^3^ [13]. The ambient air concentrations of sulfuric acid in five Brazilian anodizing plants were 7.2–2780.0 μg/m^3^, and only three of them had personal exposure concentrations ranging from 5.3 to 865.6 μg/m^3^ [5], which confirms the existence of personal exposure. Environmental monitoring levels in European companies have been determined to be between 0.0056 and 0.0300 mg/m^3^ [14], but there is a lack of data about personal exposure.

Sulfuric acid is an essential factor in electroplating. It affected not only the conductivity of the solution but also the dispersion of the electroplating solution, which is an influential factor for product yield rate [15]. However, the fixed electroplating reaction loading per pack limits the number of laborers in the process. Sulfuric acid is commonly used in these processes and industries, but it is difficult to analyze personal exposure levels due to the lack of attention and insufficient resources to perform surveys and monitoring in traditional companies.

In epidemiological studies, it would be a cost-efficient and helpful approach to develop a predictive model based on the available information and easy-to-measure factors for estimating the potential exposure levels of sulfuric acid. Therefore, this study aimed to measure the concentration of sulfuric acid exposed to workers during the electroplating process and to establish a predictive model for personal exposure to sulfuric acid concentrations in Taiwan.

## 2. Materials and Methods

### 2.1. Study Population

We obtained a list of potential candidate plants for sulfuric acid monitoring from the Taiwan Surface Finishing Association in 2018 [16]. Information about the main products, worker numbers, geographic locations, and manufacturing processes were acquired from the websites of each plant and the Department of Commerce, Taiwan Ministry of Economic Affairs [17].

Four plants were selected as representatives: an organization manufacturing small items with an integrated and automated electroplating machine (Plant A; 510 workers), an organization manufacturing small items with both an integrated and automated electroplating machine and a manual electroplating process (Plant B; 700 workers), an organization manufacturing small items with semi-automated electroplating machines (Plant C; 18 workers), and the fourth organization, manufacturing small items with integrated and automated electroplating machines (Plant D; 218 workers).

We used the data collected in Plant A to validate the predictive models built using data obtained from Plants B–D. The selected plants processed various metal parts (i.e., car, hardware, and printed circuit board parts) and conducted tin, zinc, chromium, and nickel electroplating from different products.

**Figure 1:**
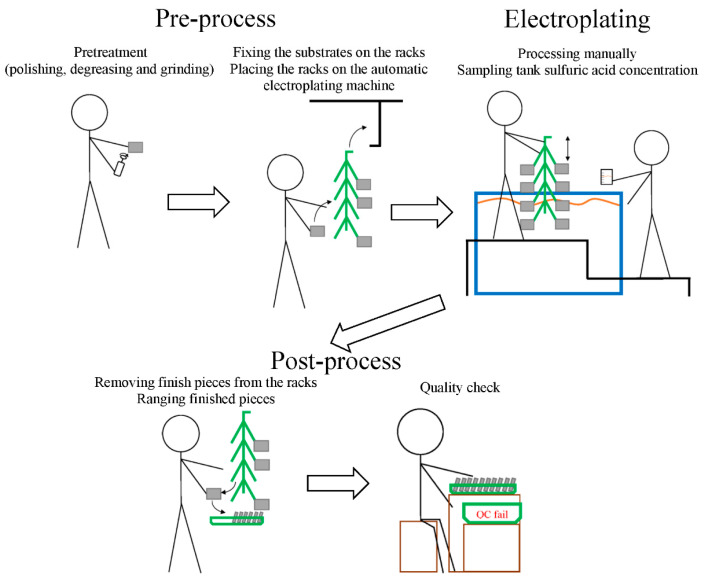
Schematic drawing of a typical electroplating process.

The schematic drawing of a typical electroplating process is shown in Figure 1. First, depending on the method, a worker pretreats the substrates by polishing, degreasing, or grinding them to improve the efficiency of electroplating. Then, the workers attach the substrates to racks and place them on an automated electroplating machine. For plants without an automated electroplating machine or with a semi-automated electroplating machine, workers have to move racks between different tanks. After automated or manual electroplating of the substrates in the tank with sulfuric acid, the workers remove the racks from the machine. The sulfuric acid mist will emit into the air due to the bubble breakage from oxygen or hydrogen assembling or water disturbed from objects moving in and out of the tanks. Finally, the electroplated substrates are removed from the racks to check the quality of the electroplating.

### 2.2. Exposure Assessment and Sampling Strategy

An industrial hygienist performed a walk-through survey at each plant to obtain information about the concentration of sulfuric acid in the tanks, tank size, operating styles, and indoor air parameters (i.e., temperature, relative humidity, atmospheric pressure, and wind speed) in the workplaces.

Because differences existed in workers’ locations and tasks both within and between plants, workers in each plant were divided into four similar exposure groups (SEGs) based on their operational processes, including pre-process, electroplating (i.e., tank quality control and rack moving), post-process (i.e., substrate collection and quality control), and office. Based on the products, plants size, process characteristics, and the number of workers at each plant, two to four workers were randomly selected to represent similar exposure levels in each of the SEGs from each plant. We collected a total of 41 personal air samples from the subjects’ exposure zones between the four plants. All participants worked the day shift (between 8:00 and 17:00, with one hour break). Personal air samples were collected from each worker during their work shift in 2018. We used air samples collected from office workers in each plant as the reference group. In addition, 8 samples were from Plant A and 11 were from each of the remaining plants (B, C, and D). We did not collect environmental samples owing to the focus on personal exposure.

During personal air sampling, the indoor air conditions were also measured. Atmospheric pressure was measured using an atmospheric pressure meter (Testo 511, Testo Ltd., Alton, UK); temperature and relative humidity were determined together using a temperature and relative humidity meter (Testo 610, Testo Ltd., Alton, UK); and wind speed was measured using an air velocity meter (TES-1340, TES Electrical Electronic Corp., Taipei, Taiwan). The accuracies (measured ranges) for these indoor air parameters were ±3.0 hPa (300–1200 hPa), ±0.5 °C (−10–50 °C), ±2.5% (0–100%), and ±0.3 m/s (0–30.0 m/s), respectively. All instruments were calibrated before use.

### 2.3. Sulfuric Acid Concentration Analysis

The method developed by the Institute of Labor, Occupational Safety and Health, Taiwan Ministry of Labor (CLA2901), was applied to analyze personal air samples of sulfuric acid. A personal sampling pump (GilAir Plus, SENSIDYNE, LP., St. Petersburg, FL, USA) was set at a flow rate of 450 mL/min and linked to a solid sorbent tube (washed silica gel, 400 mg/200 mg with a glass fiber plug (SKC 226-10-03, SKC Inc., Eighty Four, PA, USA). The sorbent tube was carried at each subject’s breathing zone to acquire sulfuric acid during a six-hour working time. Some workers wore personal protective respirators and others did not; however, these air samples only represented personal external exposure in the working environment. The obtained samples were then packed adequately and delivered to the laboratory for concentration analysis.

After the preparation process, the sulfuric acid levels in the samples were analyzed using ion chromatography (ICS-6000 HPIC, Thermo Fisher Scientific Inc., Frederick, MD, USA) with columns (AS4A-SC + AG4A-SC, Thermo Fisher Scientific Inc., Frederick, MD, USA) and conductivity detector (Dionex™ ICS-6000 CD Conductivity Detector and integrated Cell, Thermo Fisher Scientific Inc., Frederick, MD, USA, setting: 30 μS/cm). The eluent was a mixed solution of 1.8 mM Na_2_CO_3_ and 1.7 mM NaHCO_3_ at a flow rate of 1.5 mL/min. The injection loop volume was 30 μL, with a pressure of 1070 psi and a constant temperature of 24 °C. A calibration curve was established for sulfuric acid ranging from 2 to 50 μg/sample with a correlation coefficient (R) of 0.9999. The background concentration was adjusted using the average blank sorbent concentration for the batch of sorbent tubes. The limit of detection for sulfuric acid was 1 μg/sample. Before establishing the calibration curve, two blank solutions were tested to avoid contamination. A quality control check for samples was performed every 10 samples or 4 h for the quality assurance check to test the stability of the analytic system, and the recovery rate was less than 10% of the initial status.

### 2.4. Statistical Analysis

We applied the Shapiro–Wilk test to test the normality of the continuous variables, including the personal exposure concentration of sulfuric acid, temperature, relative humidity, atmospheric pressure, wind speed, tank surface, tank volume, and sulfuric acid concentration in the tank. The non-parametric Kruskal–Wallis test was used to compare differences between plants and/or processes due to abnormal distributions of these factors. For variables with significant between-group differences, the Kruskal–Wallis multiple comparison test (Dwass–Steel–Critchlow–Fligner [DSCF] method) was applied to conduct the post hoc examination. For categorical variables, Fisher’s exact test was used to compare the differences between the plants and groups. Those categorical variables with significant between-group differences were further subjected to multiple comparisons using Fisher’s exact test or the Chi-square test. Additionally, the mean, standard deviation (SD), geometric mean (GM), geometric standard deviation (GSD), median, and range were calculated as statistical descriptors.

A base-10 logarithmic transformation was applied to generate a normal distribution of sulfuric acid levels for further analysis. We used simple linear regression to identify the significant predictors for the transformed concentrations of sulfuric acid. Of the 41 samples collected, 33 samples from Plants B–D were used to develop the predictive model. We regarded air samples obtained from office workers at each plant as the reference group. The variable with/without the auto-machine was defined for plants with automatic machines or without (semi-auto or manual) machines. Multiple linear regression models were used to predict the personal levels of sulfuric acid exposure. Only parameters that generated a change greater than 10% in the adjusted r^2^ values for personal sulfuric acid levels were selected in the final model. A stepwise approach was performed to choose the parameters in the final model, where the model with the highest adjusted r^2^ was preferred. All parameters in the final model were required to have a *p*-value of <0.10. Additionally, the variance inflation factor (VIF) value was used to test the collinear relationship between predictive variables, and a VIF value of 10 was selected as the cutoff point to indicate multicollinearity of the multiple linear regression model [18,19]. Residual diagnostics were performed to ascertain whether all assumptions of linear regression models were met in the analyses. Statistical analysis software (SAS) for Windows (version 9.4) was used for the statistical analysis (SAS Institute Incorporation, Cary, NC, USA), and all tests had a significance level of 0.05 in this study.

Finally, the measured concentrations of sulfuric acid from Plant A were used to validate the predictive model developed using information obtained from other plants. The model accuracy was calculated using the sum of the square of the mean difference between the predicted and measured values (bias) and the square of the SD of the mean difference (difference = predictive level-measured level, precision) [20].

## 3. Results

**Figure 2:**
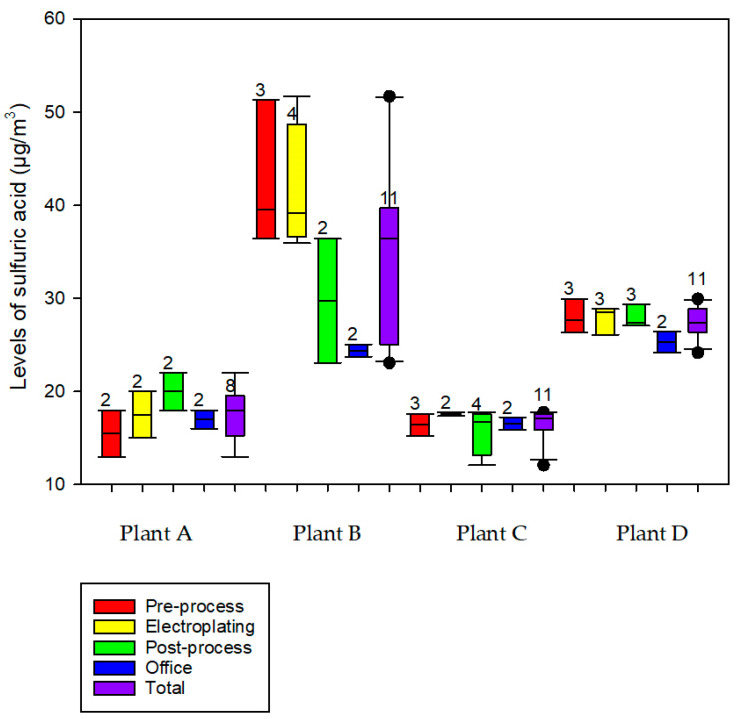
Distribution of personal sulfuric acid (μg/m^3^) by similar exposure groups between plants (sample number).

The concentrations of personal sulfuric acid measured in the four plants ranged from 12.1 to 51.7 μg/m^3^. Figure 2 shows the distribution of the SEGs among the four plants. Table 1 shows the measured levels of personal exposure to sulfuric acid and the indoor air parameters of Plants A–D. The highest level of personal sulfuric acid exposure was found in Plant B (51.7 μg/m^3^). There were significant differences in the sulfuric acid levels, temperature, relative humidity, atmospheric pressure, tank surface, tank volume, sulfuric acid concentration in the tank, and general ventilation between the four plants. Plant B had significantly higher levels of sulfuric acid and temperature but a lower mean atmospheric pressure, tank surface, tank volume, and sulfuric acid concentration in the tank than Plant A. Plant C had a significantly higher mean relative humidity and tank surface, but a lower temperature, mean atmospheric pressure, tank volume, and sulfuric acid concentration in the tank than Plant A. Plant D had a significantly higher mean temperature and relative humidity, but a lower mean atmospheric pressure, tank surface, tank volume, sulfuric acid concentration in the tank, and percentage of using general ventilation than Plant A. Additionally, Plant C had a significantly higher mean relative humidity, tank surface, tank volume, and sulfuric acid concentration in the tank, but lower sulfuric acid levels, temperature, and mean atmospheric pressure than Plant B. Plant D had significantly higher levels of sulfuric acid, temperature, atmospheric pressure, and sulfuric acid concentration in the tank, but a lower relative humidity, tank surface, tank volume, and proportion of using the general ventilation than Plant C.

The sulfuric acid levels and indoor air parameters for the different SEGs of all results are shown in Table 2. There were significant differences in wind speed and relative humidity between the various groups. The pre-process group had a significantly higher mean wind speed than the office group. Workers in the pre-process, post-process, and office had a significantly higher proportion of with/without auto-machine, but a lower proportion of using local ventilation than those in the electroplating group. The highest sulfuric acid level (mean: 51.7 μg/m^3^; range: 15.0–51.7 μg/m^3^) was found in the electroplating group but was not statistically significant.

Table 3 shows the association between indoor air parameters, workplace conditions, and 10-based logarithmically transformed levels of personal sulfuric acid in Plants B–D. The multivariate linear regression model (i.e., final mod) had a good predictive capacity (r^2^ = 0.853; adjusted r^2^ = 0.837), and the three strongest parameters were tank surface (effect estimate = −0.148 ± 0.012, *p* < 0.001), pre-process versus office (effect estimate = 0.070 ± 0.027, *p* = 0.016), and electroplating versus office (effect estimate = 0.068 ± 0.028, *p* = 0.020). The non-existence of multicollinearity in the multiple linear regression was observed because the VIF values were <10. The residual diagnostics ascertained that the residuals obeyed the Gauss–Markov conditions (residuals with zero mean, constant variance, and normal distribution).

Table 4 reveals the differences between the measured and predicted levels of personal sulfuric acid at Plant A for the established model (Model 3). The bias and precision of the predictive model were −4.98 ± 2.38 μg/m^3^ (an accuracy of 5.52 μg/m^3^) for personal sulfuric acid exposure.

**Figure 3:**
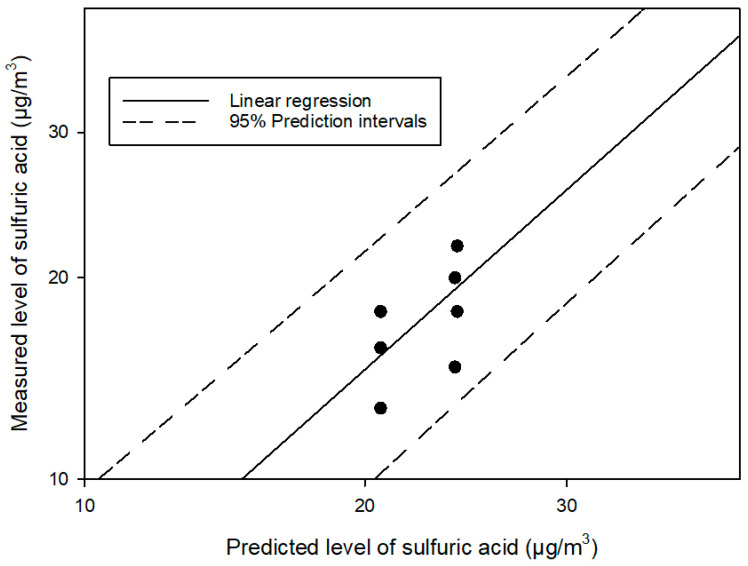
Predicted and measured levels of personal sulfuric acid (n = 8) were compared among workers at Plant A.

In Figure 3, we compared the measured level of sulfuric acids and the predicted level of sulfuric acids for Plant A. The solid line represents the linear regression between the two levels and the dashed line represents 95% prediction intervals. All results were in intervals.

## 4. Discussion

### 4.1. Main Finding

The present study revealed that, in Taiwan, laborers in a plant with electroplating processes were exposed to sulfuric acid in the range of 13.0–51.7 μg/m^3^. The exposure concentration was lower than the PEL of 1 mg/m^3^ in Taiwan. Daniels and Gunter found that personal exposure in the electroplating process of a fishing rod manufacturing plant in Denver, Colorado, was lower than the detection limit of 5 μg/m^3^ [21]. In a survey report of Greenbro industrial plating in North Carolina, personal exposure was measured to be in the range of 107–967 μg/m^3^ [22]. Another survey report at an electro-coating electroplating facility in Texas determined a range of 52–55 μg/m^3^ [23]. Our results were higher than those reported in Colorado [21], but lower than those reported in North Carolina and Texas [22,23]. However, personal exposure to sulfuric acid in electroplating workers did not exceed the PEL of 1 mg/m^3^ in either Taiwan or the USA.

Plant B had the highest exposure to sulfuric acid and was significantly higher than those of other plants. The reason could be that Plant B was a relatively larger electroplating plant. It has a plant-size auto-electroplating machine and a small room for manual electroplating, which needed workers to change the substrate racks by themselves. Especially in this small and enclosed room, the effect of engineering control was low, so a strong smell of acidity in the air in the room was present, which required further improvement to reduce the exposure.

This study demonstrated the use of field-surveyed factors and accessible information to predict personal levels of sulfuric acid exposure. Although the SO_2_ monitoring system was developed to make real-time measurements of SO_2_, it was only applied to the gaseous type of SO_2_, which is different from the particulate type of H_2_SO_4_ measured in this study. Recently, one study investigated the mechanism of sulfuric acid nucleation [24]. This research led to the further application of the real-time monitoring in the environment. Even though it can be commoditized, the results were still not adequate enough to be applied in personal exposure monitoring.

To the best of our knowledge, this was the first study to develop predictive models for personal exposure to sulfuric acid among electroplating workers in Taiwan, which had few resources to assess individual exposure among workers. The accuracy of the predictive model for sulfuric acid (i.e., 6.98 μg/m^3^) was lower than the Taiwan PEL for sulfuric acid (1 mg/m^3^), suggesting that the model is adequate for estimating potential exposure levels during preliminary workplace surveys for plants with poor working practices. Based on the guideline in the statistics, every variable needs 10–15 samples to maintain the stability of the model [25]. Therefore, a total of 33 samples was acceptable to be used for the development of a prediction model with three variables.

We also identified tank surface and process variables as significant factors for predicting sulfuric acid levels. Our study showed that a larger tank surface had a lower level of sulfuric acid exposure, which was different from the findings in a previous study that reported a positive relationship between a larger electroplating tank and a higher chemical concentration [26]. This might be due to selection bias. In our study, larger tanks implied a larger workplace, automatic machines, better workplace ventilation, and process isolation. Exposure to sulfuric acid was more critical to pre-process and electroplating when sulfuric acid was used, implying that process variables were significantly associated with predicted sulfuric acid levels because these two have stronger and closer exposure to sulfuric acid.

### 4.2. Applications

To reduce the risk of laryngeal [2,3] and lung cancers [4], clinical and histopathological changes of the nasal mucosa [5], and tooth erosion [6], OSHA defines an allowable PEL for sulfuric acid over an eight-hour day as 1 mg/m^3^. All samples collected from the four factories were below the Taiwan PEL (1 mg/m^3^). However, two samples (51.3 μg/m^3^ in the pre-process and 51.7 μg/m^3^ in electroplating) exhibited exposures higher than the OEL (0.05 mg/m^3^ in inhalable dust fractions) proposed by the ECHA [12]. In compliance with the Taiwanese and OSHA PELs, adequate control measures, good hygiene practices, and personal respirators are needed to reduce sulfuric acid levels and avoid potential health hazards among electroplating workers.

### 4.3. Strengths and Limitations

The strengths of this study included the representative sampling from different electroplating plants, detailed information collection, and the comprehensive assessment of exposure to sulfuric acid. Furthermore, statistical and logical predictive models for sulfuric acid were established with a high predictive performance. The built model can be applied to estimate the personal exposure levels of sulfuric acid for electroplating workers.

Nevertheless, some limitations exist in the present study. First, the limited number of invited and cooperative workers resulted in a relatively small sample size due to the restricted work type of the electroplating process. The limited samples could cause bias in the comparisons in average concentrations at various locations. Second, the personal sampling on a single working day may have missed variations in exposure due to the changing of production during busy and slow months. Third, the predictive capacity of sulfuric acid is limited to relatively small-scale electroplating processes rather than those in actual big corporations due to plant selection. Fourth, factors that may have affected personal exposure to sulfuric acid, such as the wind direction, field configuration, air-moving direction, and the distance between the working location and the tank, were not collected, leading to potential uncertainty when predicting individual exposure levels. Fifth, we were not allowed to obtain real-time measurements of the concentrations of sulfuric acid inside the tanks of the plants. Sixth, the final models after stepwise adjusting did not include wind speed, which is critical for predicting exposure levels. Seventh, while our models were established on silica gel, the polytetrafluoroethylene (PTFE) filter has largely replaced silica gel for capturing aerosols of sulfuric acid for measurement. The data collected using this filter should be further applied to our predictive models in future research. Finally, by using silica gel tubes instead of PTFE filters, the predictive capacity of the model was reduced owing to the sampling methodology.

## 5. Conclusions

This study reports the results of sulfuric acid exposure among electroplating workers in Taiwan. Generally, all workers were exposed to sulfuric acid levels below the Taiwan PEL. However, 2 of the 41 samples (5%) were exposed to sulfuric acid levels higher than the European OEL. Adequate control measures must be taken to decrease the exposure levels of sulfuric acid among electroplating workers in Taiwan. Despite these limitations, the developed model revealed a sufficient capacity and accuracy to estimate personal levels among workers in small-scale electroplating process plants that can be used for further epidemiological studies.

## Figures and Tables

**Table 1:** Personal exposure to sulfuric acid and parameters in different plants.

Plant	A	B	C	D	Total	*p*-Value
Sampling number (n)	8	11	11	11	41	NA
Sulfuric acid levels (μg/m^3^), M ± SD	17.5 ± 2.8	36.5 ± 9.7 ^b^	16.4 ± 1.7 ^c^	27.4 ± 1.7 ^b,d^	25.0 ± 9.8	<0.0001 ^a^
GM ± GSD(μg/m^3^)	17.3 ± 1.2	35.3 ± 1.3	16.3 ± 1.1	27.4 ± 1.1	23.3 ± 1.4	NA
Median(μg/m^3^)	18.0	36.4	17.1	27.4	23.7	NA
Range(μg/m^3^)	13.0–22.0	23.1–51.7	12.1–17.8	24.1–29.9	12.1–51.7	NA
Temperature (°C), M ± SD	26.5 ± 0.2	32.1 ± 0.6 ^b^	24.9 ± 0.7 ^b,c^	33.3 ± 0.1 ^b,c,d^	29.4 ± 3.7	<0.0001 ^a^
Relative humidity (%), M ± SD	41.9 ± 1.6	46.5 ± 6.9	83.2 ± 4.9 ^b,c^	49.9 ± 1.2 ^b,d^	56.4 ± 17.2	<0.0001 ^a^
Atmospheric pressure (mmHg), M ± SD	764.3 ± 0.2	751.9 ± 0.5 ^b^	734.4 ± 0.1 ^b,c^	736.6 ± 3.7 ^b,c,d^	745.5 ± 11.9	<0.0001 ^a^
Wind speed (m/s), M ± SD	0.8 ± 0.5	0.5 ± 0.3	0.4 ± 0.2	0.5 ± 0.3	0.5 ± 0.3	0.0906 ^a^
Tank surface (m^2^), M ± SD	1.89 ± 0	0.60 ± 0.17 ^b^	2.80 ± 0 ^b,c^	1.35 ± 0 ^b,c,d^	1.64 ± 0.84	<0.0001 ^a^
Tank volume (m^3^), M ± SD	3.96 ± 0	0.48 ± 0.13 ^b^	3.92 ± 0 ^b,c^	0.68 ± 0 ^b,c,d^	2.13 ± 1.7	<0.0001 ^a^
Sulfuric acid concentration in tank (kg/m^3^), M ± SD	0.19 ± 0	0.06 ± 0.07	0.08 ± 0 ^b,c^	0.10 ± 0 ^b,c,d^	0.10 ± 0.06	<0.0001 ^a^
With/without auto-machine, yes, n (%)	8(100)	9(81.8)	11(100)	8(72.7)	36(87.8)	0.1752 ^e^
Local ventilation, yes, n (%)	2(25)	4(36.4)	2(18.2)	0(0)	8(19.5)	0.2062 ^e^
General ventilation, yes, n (%)	8(100)	7(63.6)	9(81.8)	3(27.3) ^f,g^	24(58.5)	0.0044 ^e^

GM, geometric mean; GSD, geometric standard deviation; M, mean; n, number; NA, not available; SD, standard deviation. ^a^ The Kruskal–Wallis test was used to compare the differences between the four groups. ^b^ The Dwass–Steel–Critchlow–Fligner (DSCF) test was used to perform multiple comparisons for identifying the significant differences (*p* < 0.05) compared with those of Plant A. ^c^ The Dwass–Steel–Critchlow–Fligner (DSCF) test was used to perform multiple comparisons for identifying the significant differences (*p* < 0.05) compared with those of Plant B. ^d^ The Dwass–Steel–Critchlow–Fligner (DSCF) test was used to perform multiple comparisons for identifying the significant differences (*p* < 0.05) compared with those of Plant C. ^e^ Fisher’s exact test was used to compare the differences between the four groups. ^f^ Fisher’s exact test was used to identify the significant differences (*p* < 0.05) compared with those of Plant A. ^g^ The Chi-square test was used to identify the significant differences (*p* < 0.05) compared with those of Plant C.

**Table 2:** Exposure to sulfuric acid and indoor air parameters in different SEGs.

Group	Pre-Process	Electroplating	Post-Process	Office	Total	*p*-Value
Sampling number (n)	11	11	11	8	41	NA
Sulfuric acid levels (μg/m^3^), M ± SD	27.3 ± 11.4	29.0 ± 11.5 ^b^	21.6 ± 7.7	20.8 ± 4.4	25.0 ± 9.8	0.2241 ^a^
GM ± GSD(μg/m^3^)	25.4 ± 1.5	27.0 ± 1.5	20.4 ± 1.4	20.4 ± 1.2	23.3 ± 1.4	NA
Median(μg/m^3^)	26.3	28.5	18.0	20.9	23.7	NA
Range(μg/m^3^)	15.2–51.3	15.0–51.7	12.1–36.4	15.9–26.4	12.1–51.7	NA
Temperature (°C), M ± SD	29.4 ± 3.9	30.4 ± 3.3	28.8 ± 3.8	28.8 ± 4.2	29.4 ± 3.7	0.6104 ^a^
Relative humidity (%), M ± SD	58.6 ± 18.5	55.3 ± 14.5	58.6 ± 21.8	51.6 ± 13.8	56.4 ± 17.2	0.3791 ^a^
Atmospheric pressure (mmHg), M ± SD	746.8 ± 11	746.3 ± 12.1	743.2 ± 12.5	745.8 ± 13.8	745.5 ± 11.9	0.8360 ^a^
Wind speed (m/s), M ± SD	0.7 ± 0.3 ^b^	0.5 ± 0.4	0.5 ± 0.3	0.3 ± 0.2	0.5 ± 0.3	0.0400 ^a^
Tank surface (m^2^), M ± SD	1.66 ± 0.85	1.39 ± 0.9	1.85 ± 0.84	1.68 ± 0.83	1.64 ± 0.84	0.6503 ^a^
Tank volume (m^3^), M ± SD	2.12 ± 1.74	1.75 ± 1.74	2.43 ± 1.73	2.27 ± 1.78	2.13 ± 1.7	0.7783 ^a^
Sulfuric acid concentration in tank (kg/m^3^), M ± SD	0.09 ± 0.06	0.12 ± 0.07	0.1 ± 0.05	0.1 ± 0.06	0.1 ± 0.06	0.6902 ^a^
With/out auto-machine, yes, n (%)	11(100) ^e^	6(54.5) ^d^	11(100) ^e^	8(100)	36(87.8)	0.0019 ^c^
Local ventilation, yes, n (%)	0(0) ^e^	8(72.7) ^d^	0(0) ^e^	0(0)	8(19.5)	<0.0001 ^c^
General ventilation, yes, n (%)	8(72.7)	5(45.5)	8(72.7)	6(75)	24(58.5)	0.5025 ^c^

GM, geometric mean; GSD, geometric standard deviation; M, mean; n, number; NA, not available; SD, standard deviation. ^a^ The Kruskal–Wallis test was used to compare the differences between the four groups. ^b^ The Dwass–Steel–Critchlow–Fligner (DSCF) test was used to perform multiple comparisons for identifying the significant differences (*p* < 0.05) compared with those of the office group. ^c^ Fisher’s exact test was used to compare the differences between the four groups. ^d^ Fisher’s exact test was used to identify the significant differences (*p* < 0.05) compared with those of the office group. ^e^ Fisher’s exact test was used to identify the significant differences (*p* < 0.05) compared with the electroplating group.

**Table 3:** Associations between indoor air parameters, workplace conditions, and personal sulfuric acid (n = 33).

Model Variable	Model 1 ^a^				Model 2 ^b^			Model 3 ^c^			
	PE	SE	*p*-Value	R Square	PE	SE	*p*-Value	PE	SE	*p*-Value	PC (%)
Intercept	NA	NA	NA	NA	37.384	18.320	0.054	−1.405	0.027	<0.0001	
Temperature (°C)	0.034	0.005	<0.0001	0.643	−0.242	0.111	0.040				
Relative humidity (%)	−0.007	0.001	<0.0001	0.579	−0.022	0.013	0.110				
Atmospheric pressure (mmHg)	0.014	0.002	<0.0001	0.518	−0.040	0.019	0.052				
Wind speed (m/s)	0.227	0.117	0.062	0.108	0.812	0.413	0.063				
Tank surface (m^2^)	−0.154	0.014	<0.0001	0.806	−0.273	0.191	0.168	−0.148	0.012	<0.0001	94.5
Tank volume (m^3^)	−0.085	0.009	<0.0001	0.736	−0.353	0.195	0.084				
H_2_SO_4_ concentration in tank (kg/m^3^)	0.220	0.687	0.751	0.003	−1.111	0.906	0.234				
With/without auto-machine	−0.150	0.074	0.052	0.116	−0.155	0.130	0.246				
Pre-process vs. office	0.039	0.063	0.540	0.012	0.282	0.112	0.020	0.070	0.027	0.016	4.0
Electroplating vs. office	0.104	0.061	0.096	0.087	0.365	0.144	0.019	0.068	0.028	0.020	1.5
Post-process vs. office	−0.085	0.062	0.178	0.058	0.208	0.093	0.035				
Local ventilation	0.111	0.070	0.124	0.075	0	NA	NA				
General ventilation	−0.103	0.054	0.065	0.106	0	NA	NA				
R square	NA				0.896			0.853			
Adjusted R square	NA				0.842			0.837			

NA, not available; PE, parameter estimate; SE, standard error; PC, percentage contribution. ^a^ Simple linear regression models were used for each variable. ^b^ All variables were included in the multiple linear regression. ^c^ Three parameters of tank surface, pre-process vs. office, and electroplating vs. office (by stepwise process) were included in the multiple linear regression.

**Table 4:** Differences between predictive and measured levels of sulfuric acid for different processes.

Sampling Number	Process	Measured Level (μg/m^3^)	Tank Surface (m^2^)	Pre-Process vs. Office	Electroplating vs. Office	Predictive Level ^a^ (μg/m^3^)	Difference ^b^ (μg/m^3^)
1	Pre-process	22	1.885	1	0	24.3	2.3
2	Pre-process	18	1.885	1	0	24.3	6.3
3	Electroplating	20	1.885	0	1	24.2	4.2
4	Electroplating	15	1.885	0	1	24.2	9.2
5	Post-process	13	1.885	0	0	20.7	7.7
6	Post-process	18	1.885	0	0	20.7	2.7
7	Office	16	1.885	0	0	20.7	4.7
8	Office	18	1.885	0	0	20.7	2.7
Mean ± SD (bias ± precision)							4.98 ± 2.38
Accuracy ^c^							5.52

SD, standard deviation. ^a^ Log10(sulfuric acid) = (−1.405) + (−0.148) × tank surface + 0.070 × pre-process vs. office + 0.068 × electroplating vs. office. ^b^ Difference = predictive level—measured level. ^c^ Accuracy = $\sqrt{{bias}^{2}+{precision}^{2}}$.

## Data Availability

Data available on request due to restrictions.
